# Role of 3-Mercaptopyruvate Sulfurtransferase in the Regulation of Proliferation and Cellular Bioenergetics in Human Down Syndrome Fibroblasts

**DOI:** 10.3390/biom10040653

**Published:** 2020-04-23

**Authors:** Theodora Panagaki, Elisa B. Randi, Csaba Szabo

**Affiliations:** Chair of Pharmacology, Section of Medicine, University of Fribourg, 1700 Fribourg, Switzerland; theodora.panagaki@unifr.ch (T.P.); elisa.randi@unifr.ch (E.B.R.)

**Keywords:** trisomy, hydrogen sulfide, mitochondria, ATP

## Abstract

Down syndrome (trisomy of human chromosome 21) is a common genetic disorder. Overproduction of the gaseous mediator hydrogen sulfide (H_2_S) has been implicated in the pathogenesis of neurological and metabolic deficits associated with Down syndrome. Several lines of data indicate that an important enzyme responsible for H_2_S overproduction in Down syndrome is cystathionine-β-synthase (CBS), an enzyme localized on chromosome 21. The current study explored the possibility that a second H_2_S-producing enzyme, 3-mercaptopyruvate sulfurtransferase (3-MST), may also contribute to the development of functional deficits of Down syndrome cells. Western blotting analysis demonstrated a significantly higher level of 3-MST protein expression in human Down syndrome fibroblasts compared to cells from healthy control individuals; the excess 3-MST was mainly localized to the mitochondrial compartment. Pharmacological inhibition of 3-MST activity improved mitochondrial electron transport and oxidative phosphorylation parameters (but did not affect the suppressed glycolytic parameters) and enhanced cell proliferation in Down syndrome cells (but not in healthy control cells). The findings presented in the current report suggest that in addition to the indisputable role of CBS, H_2_S produced from 3-MST may also contribute to the development of mitochondrial metabolic and functional impairments in Down syndrome cells.

## 1. Introduction

Down syndrome, a genetic disorder caused by an aneuploidy of human chromosome 21, afflicts 1 in every 750–800 newborns annually. It occurs as a random segregation error during meiosis in the developing ovum or sperm that leads to an extra copy of the entire chromosome 21 (or part of the chromosome, resulting in partial trisomy 21) in all somatic cells. Down syndrome produces various developmental abnormalities during the fetal and neonatal life and leads to a host of pathophysiological events in adults, including various neurocognitive and metabolic impairments as well as neurodegeneration [1]. One of the biochemical hallmarks of Down syndrome is mitochondrial dysfunction which is believed to play a significant role in the pathogenesis of many of the functional defects associated with Down syndrome [2,3,4,5].

The gaseous mediator hydrogen sulfide (H_2_S) is recognized as an important endogenous regulator of various mammalian cell functions. The principal mammalian enzymes responsible for H_2_S biosynthesis are cystathionine-β-synthase (CBS), cystathionine-γ-lyase (CSE), and 3-mercaptopyruvate sulfurtransferase (3-MST) [6,7,8]. CBS—localized on chromosome 21—is overexpressed in Down syndrome cells and tissues [reviewed in 5]. Recent studies on Down syndrome fibroblasts, using pharmacological inhibition of CBS, as well as genetic silencing of CBS, indicated that the metabolic inhibition seen in Down syndrome cells, is at least, in part, caused by the overproduction of H_2_S by CBS [9]. The excess intracellular H_2_S was shown to inhibit mitochondrial Complex IV activity to produce a reversible inhibition of mitochondrial electron transport and an impairment of aerobic ATP generation [9].

The current study explored the potential modulatory role of a second H_2_S-producing enzyme, 3-MST, in the regulation of the proliferation and cellular bioenergetics of Down syndrome fibroblasts. The results presented in the current paper indicate that 3-MST accumulates in the mitochondria of Down syndrome cells and contributes to the increase in cellular H_2_S levels. Moreover, the data indicate that 3-MST-derived H_2_S contributes to the suppression of mitochondrial function in Down syndrome cells.

## 2. Materials and Methods

### 2.1. Materials

The cell proliferation ELISA 5-bromo-2′-deoxyuridine (BrdU) kit was purchased from Roche Diagnostics Ltd. (Sigma–Aldrich Chemie Gmbh: Munich, Germany). The β -actin (8H10D10) mouse monoclonal antibody (#3700), the Tom20 (D8T4N) rabbit monoclonal antibody (#42406), the ELISA Lysis Buffer (1X), the protease/phosphatase inhibitor cocktail (100X), and horseradish peroxidase (HRP)-linked secondary antibodies against the corresponding species IgG of the primary antibodies were obtained from Cell Signaling Technology (Bioconcept AG: Allschwill, Switzerland). The 3-MST rabbit polyclonal antibody (ab85377) and the Amersham ECL Prime Western blotting detection reagent kit were obtained from Abcam PLC (Cambridge, UK) and GE Healthcare Life Sciences (Sigma–Aldrich Chemie Gmbh: Munich, Germany), respectively. The mitochondrial isolation kit MITOISO2, the fluorescent H_2_S probe 7-azido-4-methylcoumarin (AzMC) [10], the fluorescent polysulfide probe (*E*)-2-(3-(6-(2-hydroxyethylamino)naphthalen-2-yl)-3-oxoprop-1-enyl)-3,5-dimethoxybenzaldehyde (P3) [11], bovine serum albumin (BSA), and lactalbumin hydrolysate were purchased from SigmaAldrich Chemie Gmbh (Munich, Germany). All other materials and reagents for cell culture and Western blotting were obtained from Thermo Fisher Scientific (Basel, Switzerland), unless otherwise stated.

### 2.2. Cell Culture

Human dermal fibroblasts from healthy control subjects and individuals with Down syndrome were obtained from LGC Standards (Wesel, Germany), the Coriell Institute (Camden, NJ, USA), and the Jérôme Lejeune Institute (Paris, France), as summarized in Table 1. Fibroblasts were cultured in Advanced Dulbecco’s Modified Eagle Medium/nutrient mixture F-12 (DMEM / F-12, 1:1; 1X) supplemented with 10% heat-inactivated fetal bovine serum (FBS), 10 UI/mL of penicillin, 100 µg/mL streptomycin, 2 mM Glutamax™, and 0.1% lactalbumin hydrolysate (hereafter referred to as complete growth medium). Cells were maintained at 37 °C in a humidified incubator with 5% CO_2_ and 95% air. Cells were sub-cultured when 80–90% confluent and seeded at a ratio of 1:4. When passaged, viable cells were counted and seeded at the desired cell density for the assays using an Automated Cell Counter R1 (Olympus: Volketswil, Switzerland). The culture medium was replaced every one to two days.

### 2.3. Cell Treatments

The 3-MST inhibitor 2-[(4-hydroxy-6-methylpyrimidin-2-yl)sulfanyl]-1-(naphthalen-1-yl)ethan-1-one (HMPSNE) [12] was purchased from MolPort (Riga, Latvia). The inhibitor was reconstituted in pure anhydrous DMSO to a stock concentration of 0.5 M. The stock solution was aliquoted and stored at −80 °C until further use. All aliquots were thawed once and serially diluted in the complete growth medium to final working concentrations of 1–10 µM.

### 2.4. Cell Proliferation Assay

The cell proliferation ELISA BrdU (colorimetric) assay was performed in Corning^®^ Costar^®^ TC-treated flat-bottomed 96-well plates. Diploid and 21-trisomic fibroblasts were seeded at a density of 1 × 10^4^ cells per well in a total of 100 µL for 2 h. Cells were subsequently treated with 0, 1, 3, and 10 µM HMPSNE in complete growth medium for 24 h. Following the treatments, fibroblasts were incubated with 10 µM BrdU labelling solution for 4 h at 37 °C in a humidified incubator with 5% CO_2_ and 95% air. The assay is based on the principle that the pyridine analogue BrdU is incorporated, in place of thymidine, into the newly synthesized DNA strands of proliferating cells. Following mild fixation and DNA denaturation, BrdU incorporation was detected by immune-peroxidase staining and a subsequent colorimetric substrate reaction as per the manufacturer’s protocol. Plates were read at 450 and 690 nm (reference wavelength) using an Infinite^®^ 200 PRO micro-plate reader (Tecan Group Ltd.: Männedorf, Switzerland). Developed color and absorbance values reflect the amount of DNA synthesis and thereby the number of proliferating cells in the respective micro-cultures.

### 2.5. Quantification of H_2_S Levels in Live Cells

The assay was performed in Thermo Fisher Nunc^®^ 96-well black, optical-bottom plates. Following treatment with the 3-MST inhibitor or its vehicle (as described in the previous subsection), fibroblasts were incubated with 10 µM of AzMC for 1 h at 37 °C in a humidified incubator with 5% CO_2_ and 95% air. The fluorescence signal of the AzMC probe was read with an Infinite^®^ 200 PRO microplate reader at λ_excitation_ = 365 nm and λ_emission_= 450 nm. A rise in the signal corresponds to an increased reduction of the aromatic azide moiety in the presence of H_2_S and the production of the fluorescent 7-amino-4-methylcoumarin. It is therefore proportional to the endogenous H_2_S levels in the respective micro-cultures. A second, structurally different H_2_S fluoroprobe (P3) was also employed to confirm the findings obtained with AzMC. Cells were incubated with 10 µM of P3 for 1 h at 37 °C in a humidified incubator with 5% CO_2_ and 95% air, washed twice with pre-warmed 1X PBS, and then their fluorescent signal was detected with the Infinite^®^ 200 PRO microplate reader at λ_excitation_ = 375 nm and λ_emission_ = 505 nm. The AzMC and P3 fluorescent signals were both corrected to the total protein content of each condition and background (cell auto-fluorescence).

### 2.6. Measurement of Mitochondrial Respiration

Extracellular flux (XF) analysis was employed for real-time quantification of oxygen consumption owing to ATP turnover, proton leak, and maximal respiratory capacity in live cells as described [13,14]. The assay was formatted in Seahorse XF24 cell-culture microplates, where diploid and 21-trisomic fibroblasts were seeded at a density of 2 × 10^4^ cells per well in a total of 200 µL for 2 h. Cells were subsequently treated as described above for 24 h, and mitochondrial respiration was assessed as previously described [13,14].

### 2.7. Sample Preparation for Whole-Cell Protein Extraction

Cells (0.5 × 10^6^) were grown in Corning^®^ 25 cm^2^ rectangular cell culture flasks until ~80–90% confluency. Thereafter, cells were washed once with ice-cold 1X PBS and harvested in pre-cooled 1X ELISA lysis buffer previously supplemented with protease/phosphatase inhibitor cocktail (1X). Following a freeze/thaw cycle, whole-cell lysate was collected and sonicated for 5 min (30 s ON/30 s OFF) in an ultrasonic water-bath. Total protein was extracted by centrifugation at 16,000× *g* at 4 °C for 15 min. Pierce™ Coomassie Plus Bradford protein assay was conducted to quantify the protein concentration of the samples.

### 2.8. Sample Preparation for Mitochondrial and Cytosolic Protein Extractions

Enriched mitochondrial fractions were prepared with the MITOISO2 kit per the manufacturer’s protocol. Briefly, 3 × 10^6^ cells were grown in Corning^®^ 150 cm^2^ rectangular cell culture flasks until ~80–90% confluency. Cells were then trypsinized and collected by centrifugation at 600× *g* at 4 °C for 5 min. The cell pellet was subsequently washed twice in ice-cold 1X PBS and re-suspended to a uniform suspension in 100 µL of the provided lysis buffer per 2 × 10^6^ cells. Cell lysis was conducted by vigorously vortexing the suspension every minute for a total of 5 min. Mitochondria were then stabilized by the addition of 1X extraction buffer. The suspension was centrifuged at 600× *g* at 4 °C for 10 min to initially pellet nuclei and cell debris. The supernatant was collected and re-centrifuged at 10,000× *g* at 4 °C for 10 min to pellet mitochondria. The new supernatant was enriched in cytosolic fractions and collected in a new micro-tube while the mitochondrial-enriched pellet was reconstituted in 1X storage buffer. Following collection, Pierce™ Coomassie Plus Bradford protein assay was conducted to estimate the protein concentration of both fractions, which were subsequently processed for western blotting.

### 2.9. Western Blotting

Protein samples from whole-cell lysate, mitochondrial- or cytosolic-enriched extractions (5 µg) were separated on Bolt™ 4–12% gradient Bis—Tris gel and blotted onto nitrocellulose membranes, as per our previously published protocol [15]. Blots were blocked in 5% *w/v* skimmed milk for 1 h at room temperature and probed with the primary antibodies against 3-MST, Tom20, and β -actin overnight at 4 °C with gentle agitation. The primary antibodies were diluted in 5% BSA in 1X TBS with 0.05% Tween^®^ 20 (TBS–T; pH 8) at 1:100, 1:1000, and 1:2000. Following the primary antibody incubation, blots were assayed for chemiluminescent detection of the proteins of interest, as previously described [15]. The Azure 300 Chemiluminescent Imaging System (Azure Biosystems: Dublin, CA, USA) and Image J (National Institutes of Health: Bethesda, MA, USA) were used to capture the image chemiluminescent bands and to perform densitometric analysis. We used β-actin as a loading control to which the relative peak intensities of the examined markers were normalized.

### 2.10. Statistics

The results were expressed as the mean ± standard error of the mean (SEM) of at least three independent experiments or eight independent pairs of diploid and 21-trisomic human fibroblasts. Differences among means were considered significant when *p* < 0.05. Two-way ANOVA, followed by post-hoc Bonferroni’s multiple-comparison t-test, was used to identify differences among groups of treated and untreated conditions. Alternatively, an unpaired two-sample t-test was used to identify differences between diploid and aneuploid cells. Statistical calculations were performed using GraphPad Prism 8 (GraphPad Software Inc.: San Diego, CA, USA).

## 3. Results

### 3.1. Down Syndrome Fibroblasts Overexpress 3-MST, which Accumulates in the Mitochondria

**Figure 1:**
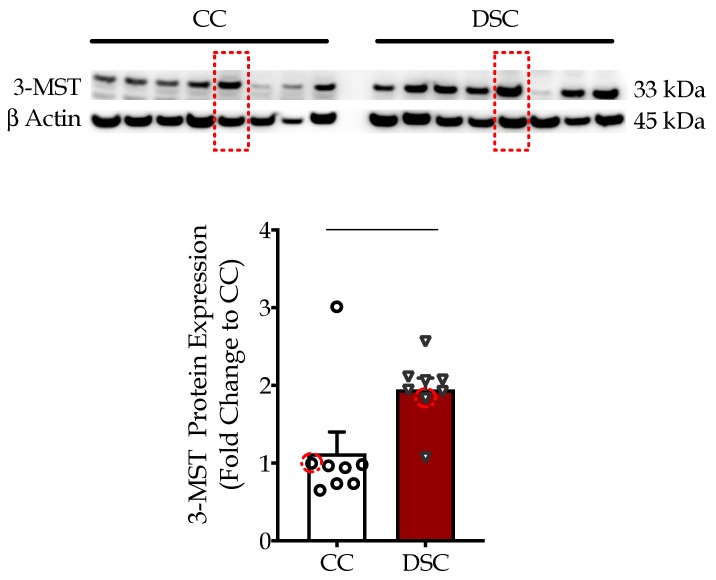
Down syndrome cells (DSC) exhibit significantly higher 3-MST protein expression compared to control cells (CC) as shown by Western blotting. We used β-actin as a loading control. Each bar represents the mean ± SEM of 8 human healthy control fibroblasts and 8 human Down syndrome fibroblasts (summarized in Table 1). ** *p* < 0.01. Similar to our previous study [9], the normal fibroblast cell line Detroit 551 and the Down syndrome fibroblast Detroit 539 were compared in the subsequent functional studies. 3-MST and β-actin expression in these two selected cell lines are indicated with red dotted squares.

We initially quantified the expression levels of 3-MST in eight human fibroblast cell lines from different healthy subjects and in eight human fibroblast cell lines obtained from different individuals with Down syndrome. Western blotting analysis demonstrated that 3-MST expression was approximately 50% higher in 21-trisomic fibroblasts than in the control fibroblasts (Figure 1).

**Figure 2:**
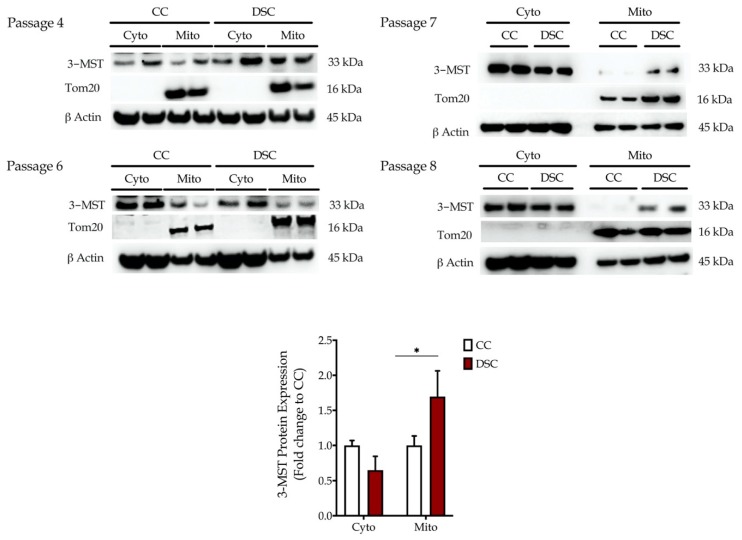
3-MST accumulates in the mitochondria of Down syndrome cells (DSC) compared to healthy control cells (CC) as shown by Western blotting. Top panels: chemiluminescence images of 3-MST protein expression in cytosolic (Cyto)- and mitochondrial (Mito)-enriched fractions of the selected healthy control fibroblast (Detroit 551; CC) and the Down syndrome fibroblast (Detroit 539; DSC). Experiments using four different cell passages are shown. The Western-blot analysis utilized β-actin as a loading control. The outer mitochondrial membrane transport protein Tom20 served as a control for the purity of the mitochondrial-enriched fractions. Bottom graph: statistical analysis of the healthy control fibroblast (Detroit 551; CC) and the Down syndrome fibroblast (Detroit 539; DSC). * *p* < 0.05.

Similar to our previous study [9], the normal fibroblast cell line Detroit 551 and the Down syndrome fibroblast Detroit 539 were compared in the subsequent functional studies. First, we wished to delineate the subcellular localization of 3-MST. 3-MST protein levels were quantified in the extracted cytosolic- and mitochondrial-enriched fractions (Figure 2). Down syndrome fibroblasts exhibited a markedly higher mitochondrial 3-MST content compared to mitochondrial 3-MST in the healthy control cells. Thus, the aberrant mitochondrial 3-MST in Down syndrome cells may not only reflect a rise in the absolute expression levels, but also a translocation from the cytoplasm into the mitochondria. In fact, we noted a trend (*p* = 0.06) of lower cytosolic expression of 3-MST in Down syndrome fibroblasts compared to healthy control cells (Figure 2).

### 3.2. HMPSNE Inhibits H_2_S Production and Restores the Cellular Proliferation Rate in Down Syndrome Cells

**Figure 3:**
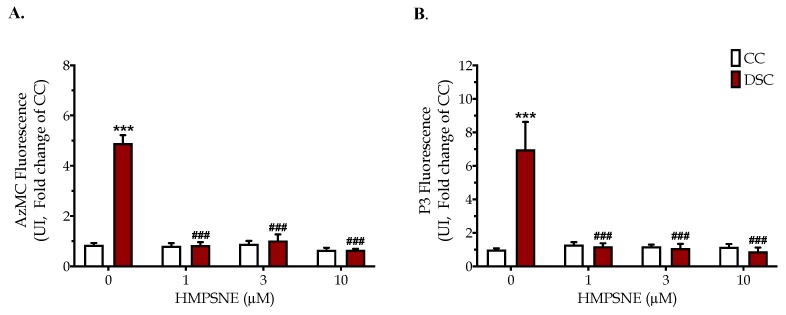
The 3-MST inhibitor HMPSNE suppresses H_2_S levels in Down syndrome cells. Fluorescence values of the H_2_S probe AzMC (**A**) and P3 (**B**) are shown in healthy control Detroit 551 fibroblasts (CC) and in Down syndrome Detroit 539 (DSC) fibroblasts under baseline conditions and when treated with 1, 3 or 10 µM HMPSNE for 24 h. Each bar represents the mean ± SEM of 4 independent experiments. *** *p* < 0.001 indicates a significant increase in fluorescence in DSCs vs. CCs; ^###^ *p* < 0.001 indicates a significant inhibitory effect of HMPSNE on cellular H_2_S levels in DSCs.

Consistent with the increased 3-MST expression, 21-trisomic fibroblasts exhibited increased levels of H_2_S, as evidenced by the increase in the fluorescent AzMC and P3 signals (Figure 3). The HMPSNE-associated normalization of the endogenous H_2_S levels in Down syndrome cells was already complete at the lowest (1 µM) inhibitor concentration used.

**Figure 4:**
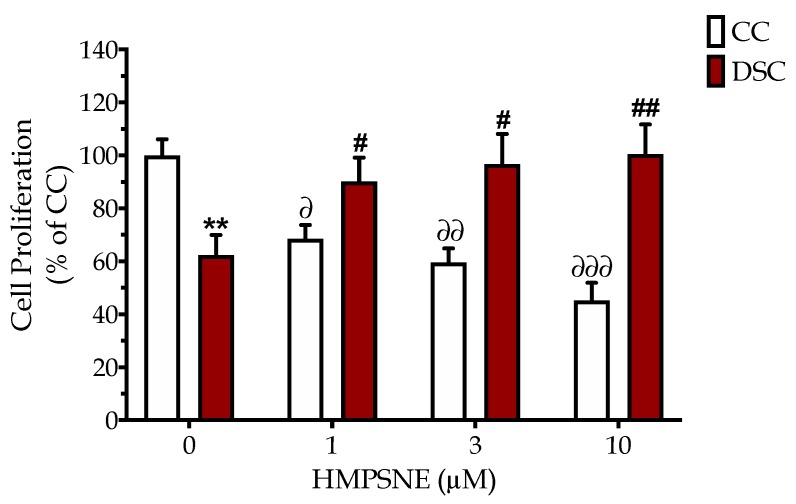
The 3-MST inhibitor HMPSNE concentration-dependently restores the cell proliferation rate in Down syndrome cells. BrdU incorporation assay was used to quantify cell proliferation of normal Detroit 551 (CC) and Down syndrome Detroit 539 (DSC) fibroblasts under baseline conditions and when treated with 1, 3 or 10 µM HMPSNE for 24 h. Each bar represents the mean ± SEM from at least 3 independent experiments. ** *p* < 0.01 indicates that baseline cell proliferation was significantly lower in DSCs than in CCs; ^∂^ *p* < 0.05 ^∂∂^ *p* < 0.01 and ^∂∂∂^ *p* < 0.001 indicate the significant inhibitory effects of HMPSNE on the proliferation of CCs; ^#^ *p* < 0.05 and ^##^ *p* < 0.01 indicate the significant stimulatory effect of HMPSNE on the proliferation of DSCs.

The basal proliferation rate of Down syndrome cells was slower than the corresponding rate of the healthy control cells (Figure 4). Inhibition of 3-MST with HMPSNE reduced the proliferation rate of the control cells; however, the same concentrations of the 3-MST inhibitor concentration-dependently increased the proliferation rate of Down syndrome cells and at the highest HMPSNE concentration used (10 µM), restored it to levels of the healthy control cells (Figure 4).

### 3.3. HMPSNE Normalizes Cellular Bioenergetics in Down Syndrome Cells

**Figure 5:**
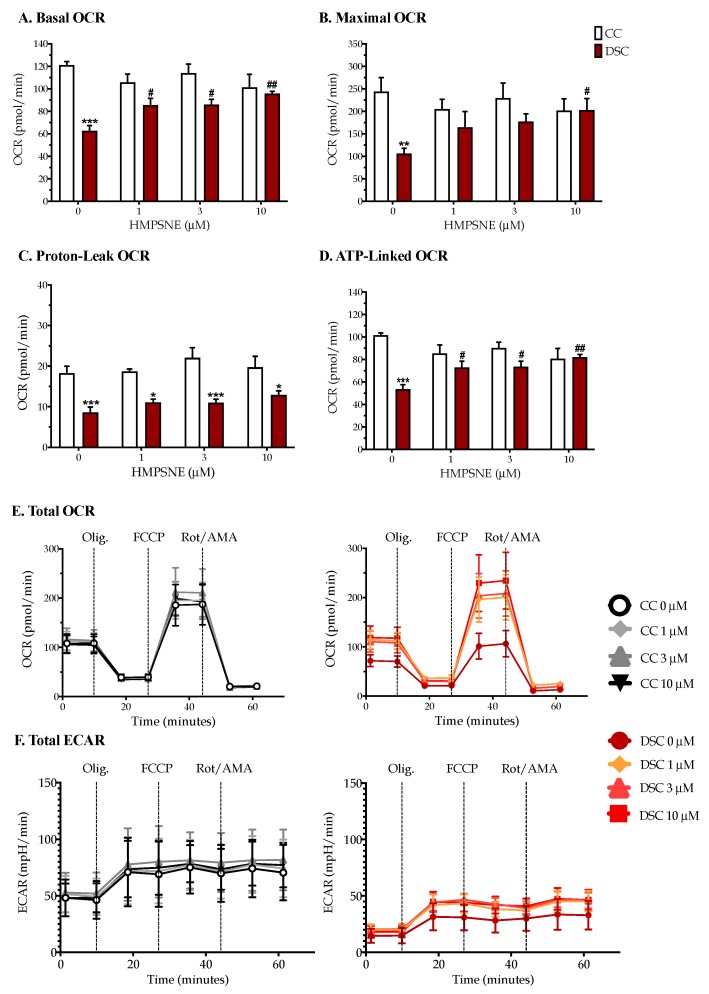
The 3-MST inhibitor HMPSNE normalizes the suppressed oxidative phosphorylation in DS cells. (**A**) Basal OCR; (**B**) Maximal OCR; (**C**) Proton-Leak OCR; (**D**) ATP-Linked OCR; (**E**) Total OCR; (**F**) Total ECAR. Cellular bioenergetics was quantified with the Seahorse extracellular flux analyzer. Each bar and line represent the mean ± SEM from at least 3 independent experiments of the selected normal Detroit 551 (CC) and Down syndrome Detroit 539 (DSC) fibroblasts under baseline conditions and when treated with 1, 3 or 10 µM HMPSNE for 24 h. * *p* < 0.05, ** *p* < 0.01, and *** *p* < 0.001 indicate that basal metabolic parameters were significantly lower in DSCs than in CCs; ^#^ *p* < 0.05 and ^##^ *p* < 0.01 indicate a significant stimulatory effect of HMPSNE in DSCs compared to the values in the absence of the 3-MST inhibitor.

The low basal oxygen consumption rate (OCR) and extracellular acidification rate (ECAR) of Down syndrome cells (compared to healthy control cells) (Figure 5) indicates that Down syndrome cells exhibit both an impaired mitochondrial respiration (aerobic bioenergetic function) and suppressed glycolysis (anaerobic bioenergetic function). Pharmacological inhibition of 3-MST with HMPSNE did not exert any significant effect on the bioenergetic parameters of healthy control cells (although in some cases a trend of a decrease was noted) (Figure 5B,D). In contrast, in Down syndrome cells, HMPSNE concentration-dependently increased the basal cellular OCR, the maximal OCR, and the ATP-linked OCR (Figure 5A,B,D). In the presence of 10 µM HMPSNE, the mitochondrial energetic parameters of Down syndrome cells were comparable to those of the healthy control cells. In contrast to its effect on oxidative phosphorylation-related bioenergetic parameters (Figure 5E), the 3-MST inhibitor did not significantly affect the glycolytic parameters in control or Down syndrome cells, although at the highest concentration used, a trend of an inhibitory effect was noted (Figure 5F).

## 4. Discussion

According to the “Kamoun Hypothesis”, originally proposed by French biochemist and physician Pierre Kamoun, in Down syndrome, a toxic overproduction of H_2_S occurs, which, in turn, creates a metabolic cell poisoning, at least in part through inhibition of mitochondrial Complex IV [16,17]. The hypothesis was originally developed based on clinical studies showing that in individuals with Down syndrome, there is a marked elevation observed in circulating and urinary H_2_S metabolites [18,19]. Because CBS is located on chromosome 21, it was naturally assumed that the cause of the elevation of H_2_S levels was a “gene dosage” effect: the extra chromosome encodes extra CBS enzyme in the cells and tissues of Down syndrome individuals. Indeed, the upregulation of CBS has been well documented in various cells and tissues of animals or human subjects with Down syndrome [9,20,21,22,23,24]. Our group recently generated direct experimental evidence in support of the Kamoun hypothesis: in Down syndrome fibroblasts (Detroit 531 cells), we demonstrated that pharmacological inhibition of CBS (using the small molecule aminooxyacetate) or silencing of CBS restores the suppressed Complex IV activity to near-normal control levels and improves mitochondrial electron transport and cell proliferation [9].

The results of the current study, however, indicate that in addition to CBS, a second H_2_S-producing enzyme, 3-MST, may also contribute to the cellular bioenergetic disturbances in Down syndrome fibroblasts. Although 3-MST is not localized to chromosome 21 (but is localized to chromosome 22), our data, generated using Down syndrome fibroblasts from eight different individuals of different ages and genders (Table 1), demonstrate that 3-MST protein levels are elevated in the Down syndrome cells (Figure 1). Subcellular localization studies show that most of this elevation is due to an increase in the mitochondrial accumulation of this enzyme (Figure 2).

Emerging data demonstrate that the dysregulation of gene expression in Down syndrome goes well beyond the “gene dosage effect” (i.e., upregulation of multiple genes encoded on chromosome 21). In fact, approximately 80% of the mRNA that is differentially expressed in Down syndrome cells is not localized on chromosome 21, but on the other chromosomes [25,26,27,28,29]. These mRNAs, for most part, are upregulated, but in some instances, downregulation was also noted. There are significant cell and tissue differences in these gene expression dysregulation patterns [26]. The exact molecular mechanisms underlying the changes in non-chromosome-21-encoded mRNA expression are incompletely understood; it has been hypothesized that a global cell stress response and/or a subsequent activation of various compensatory mechanisms may be contributing factors [27]. In the current project, we focused on the expression of the 3-MST protein (and not mRNA); a prior RNAseq analysis conducted by Sullivan and colleagues did not indicate significantly higher mRNA levels for 3-MST in Down syndrome cells compared to healthy control cells (mRNA expression in Down syndrome cells was approximately 108% of that in the control) [25]. However, in another study (in Down syndrome thymus tissue) the micro-RNA miR-193b-3p was found to be downregulated, which would predict an upregulation of the 3-MST protein [30]. The above two findings are not necessarily in conflict: changes in micro-RNA expression often, but not always or necessarily, produce detectable changes in the levels of the corresponding mRNA [31]. We hypothesize that Down syndrome cells upregulate the 3-MST protein through the regulation of mRNA stability, and/or through various transcriptional mechanisms that may become activated in response to the presence of the additional 21^st^ chromosome in the cells, and/or through post-translational mechanisms (i.e., inhibition of 3-MST protein degradation), and/or through an increased transport of 3-MST into the mitochondria. Indeed, prior studies have shown that Down syndrome cells have significant disturbances in transcriptional and protein degradation and processing mechanisms, as well as in mitochondrial protein accumulation [32,33,34,35]. The above hypotheses describe conceivable potential mechanisms. Nevertheless, future experimental work will be necessary to delineate the molecular mode(s) of 3-MST protein accumulation in Down syndrome.

**Figure 6:**
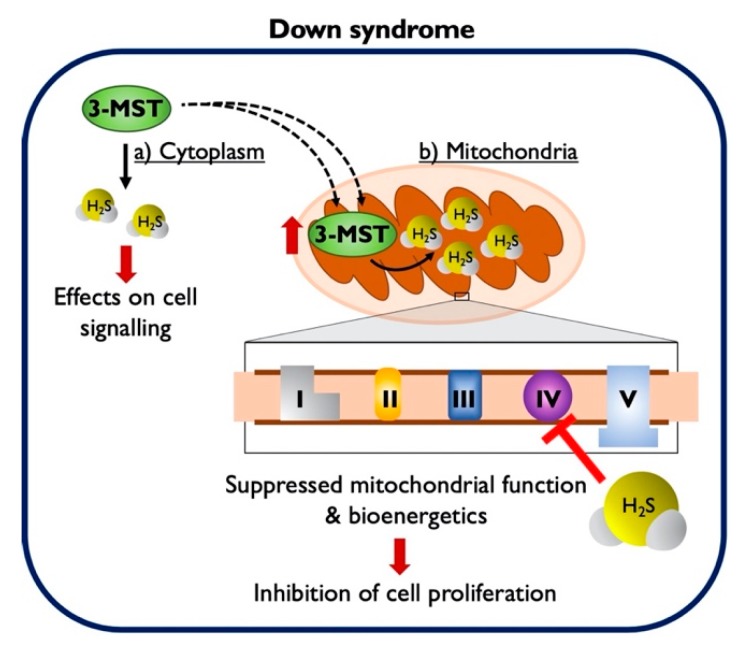
The 3-MST protein is upregulated in Down syndrome and contributes to cell dysfunction. The excess 3-MST is transported into the mitochondria. The subsequent overproduction of H_2_S suppresses mitochondrial electron transport and causes a cellular bioenergetic deficit, which is evidenced, amongst other responses, by a suppression of the cellular proliferation rate. Pharmacological inhibition of 3-MST with HMPSNE (not shown in this scheme) corrects H_2_S overproduction and improves bioenergetic parameters, resulting in the stimulation of Down syndrome cell proliferation.

Although the exact molecular mechanisms remain to be further elucidated, the fact remains that Down syndrome fibroblasts contain approximately two times more 3-MST than control cells (Figure 1), and most 3-MST is localized in the mitochondrial compartment, while the cytosolic 3-MST content tends to decrease (Figure 2). What, then, is the functional role of this enzyme in the regulation of bioenergetics and proliferation of Down syndrome cells? To address this question, we employed the recently discovered 3-MST inhibitor HMPSNE, which, to date, is the most potent and most selective pharmacological inhibitor of this enzyme [10,36]. HMPSNE has been successfully employed in cell-based studies in various cell types, at concentrations similar (or higher) [14,37,38,39] than those used in the current project. Pharmacological inhibition of 3-MST in healthy control fibroblasts only had a slight effect on cellular bioenergetic parameters, but it reduced their proliferation rate. We hypothesize that the growth-inhibitory effect of HMPSNE is related to the inhibition of the physiological stimulatory effect of H_2_S on various proliferative signaling pathways; these effects are, presumably, linked to the cytosolic (rather than the mitochondrial) component of 3-MST. (In fact, it is well known that physiological concentrations of H_2_S play a role in the maintenance of various cell proliferation pathways, for instance, through the sulfhydration of Akt [40,41,42,43]). In contrast, in Down syndrome fibroblasts (which contain markedly elevated mitochondrial 3-MST), the 3-MST inhibitor enhanced mitochondrial respiration and various parameters of basal and stimulated mitochondrial oxygen consumption, consistent with the classic hypothesis [5,16] that the mitochondria of Down syndrome cells are under a tonic H_2_S-mediated suppression of mitochondrial function. In Down syndrome cells (in stark control to the healthy control cells), 3-MST inhibition caused a stimulation of the cell proliferation rate. We hypothesize that the improved bioenergetics of Down syndrome cells after HMPSNE treatment may, in turn, contribute to the stimulation of the cellular proliferation rate (Figure 6). H_2_S clearly has the capacity to suppress mitochondrial function [5,8] (as previously shown for CBS-derived H_2_S in Down syndrome [9]). Nevertheless, 3-MST is a multifunctional enzyme with multiple regulatory roles (e.g., by influencing cellular redox processes) [7,8,36]. Thus, the mechanism proposed in Figure 6 is a feasible one, but perhaps not the only one that is possible. Further experiments, for example, attempting to reverse the functional effect of HMPSNE with a chemical H_2_S donor, might be useful to further test the exact mode involved in the functional effect of 3-MST in Down syndrome cells. We recently conducted similar studies with the H_2_S donor GYY4137 in Down syndrome cells, and these data demonstrated that indeed, excess H_2_S in normal control cells can lead to a suppression of mitochondrial bioenergetics and a suppression of cell proliferation [9]. Moreover, at least in the case of CBS-derived H_2_S, the effect of CBS inhibition or CBS silencing can be reversed by GYY4137 in Down syndrome cells [9], pointing to a direct involvement of H_2_S in the bioenergetic dysfunction associated with Down syndrome.

Which enzyme, then, is more important in the suppression of mitochondrial function in Down syndrome, CBS or 3-MST? When comparing the extent of the beneficial effect observed with the CBS inhibitor aminooxyacetate (or CBS silencing) in a prior study [9] with the effect of HMPSNE in the current study, we can conclude that CBS inhibition exerted more robust effects. However, surprisingly, both approaches produced a comparable (complete or near-complete) inhibition of cellular H_2_S levels (at least, as detected by the fluorescent H_2_S dyes, e.g., AzMC, used in our laboratory). We expected that since both enzyme systems contribute to the total cellular H_2_S production, inhibition of either CBS or 3-MST would produce a partial inhibition of the cellular H_2_S levels (as opposed to the complete or near-complete inhibitions observed). We do not have a clear explanation of this unexpected finding, but it is possible that the sensitivity or the cell permeation of the fluorescent dye used may play a role.

Another surprising finding was the lack of HMPSNE’s effect on glycolytic parameters (in normal cells or in Down syndrome cells). Prior studies in various cell types have indicated that H_2_S regulates key glycolytic enzymes [44,45]. Moreover, 3-MST silencing in adipocytes was recently reported to increase the extracellular acidification rate [46], while in endothelial cells, 3-MST silencing or HMPSNE decreased various glycolytic parameters [39]. In murine colon cancer cells, HMPSNE exerted a bell-shaped effect on glycolytic parameters (increases at the lowest inhibitor concentration used and decreases at higher concentrations) [14]. Different cell types in culture may rely to a different extent on oxidative phosphorylation vs. glycolysis, and the biochemical mechanisms regulating these processes may also be cell-type different, which may explain the lack of HMPSNE’s significant effect on glycolysis in the current study.

Although both CBS and 3-MST are superficially viewed as “mammalian H_2_S-producing enzymes”, there are important differences between them, not only with regards to their cellular and subcellular localization, substrates, and enzymatic regulation (different substrates, different co-factors), but also the product(s) of the reactions they catalyze [5,6,7]. Importantly, while the product of CBS is primarily “free” H_2_S (which, in turn, dissociates into hydrosulfide anion), 3-MST is primarily considered a source of polysulfides. Polysulfides are biologically active sulfide species, which exert their effects primarily through posttranslational modification of various enzymes (sulfhydration) [45,47]. The combination of increased CBS and increased 3-MST in Down syndrome cells would predict increased H_2_S, as well as increased polysulfide levels, which, in turn, would be expected to produce post-translational modifications in multiple proteins in all cellular compartment (mitochondria, cytosol, cell membranes, etc.). Characterization of the sulfhydrated proteins in cells and tissues of individuals with Down syndrome (to establish the “Down syndrome sulfhydrome”) and delineation of the alterations in the function of the sulfhydrated proteins remain to be conducted in future studies. It is also important to mention that both CBS and 3-MST have important roles in the regulation of cellular redox homeostasis: CBS activity affects upstream steps in the transsulfuration process, which, in turn, affects cellular glutathione levels (an important antioxidant) [8]. Moreover, in addition to its role as a H_2_S/polysulfide-producing enzyme, 3-MST is also an antioxidant enzyme and one whose activity is regulated by the cellular redox status [48]. The alterations in oxidant/antioxidant balance in Down syndrome are complex. Importantly, not only are various pro-oxidant processes upregulated in Down syndrome, but so is antioxidant superoxide dismutase (SOD), which is also encoded on chromosome 21 [49]. Nevertheless, the net effect of these various alterations is increased intracellular pro-oxidant levels in Down syndrome cells and tissues [49,50]. How CBS and/or 3-MST levels modulate the intracellular redox homeostasis is currently unclear and remains to be investigated in future studies.

Irrespective of the source of the excess H_2_S in Down syndrome (CBS, 3-MST or both), so far, most of the studies focusing on the “Kamoun Hypothesis” rely on in vitro data or clinical (observational) studies. Thus, the in vivo experimental testing of the “Kamoun Hypothesis” (e.g., in animal models of Down syndrome) remains to be conducted. There are recent in vivo data from the Herault group demonstrating that Down syndrome mice (i.e., mice with trisomy of the mouse chromosome fragment that carries murine CBS as well as several other genes) develop neurological deficits, and inactivation of CBS in these mice reverses the functional deficits [29]. Moreover, forced overexpression of CBS in mice has been shown to produce disturbances in serotonin and dopamine pathways in the brain of mice [51] and to cause neurobehavioral deficits in some (but not all) experiments conducted to date [29,52]. With respect to 3-MST, there are also in vivo studies showing that both its deletion and its overexpression can impair various neurobehavioral parameters in mice [53,54]. In addition, ethylmalonic encephalopathy, an autosomal recessive disease which is associated with neurological impairment is, at least in part, due to the excessive accumulation of H_2_S; in this instance this is caused by ETHE1 mutations, which decrease the clearance of H_2_S due to the downregulation of mitochondrial sulfur dioxygenase [55]. These data, taken together, support the view that excessively high levels of H_2_S in the central nervous system exert detrimental effects on neurological functions and should encourage additional in vivo studies to further test the “Kamoun Hypothesis” in terms of short-term neurological defects, as well as long-term metabolic alterations and perhaps in terms of later-stage neurodegenerative processes as well.

## 5. Conclusions

In conclusion, the results presented in the current study demonstrate that Down syndrome fibroblasts express increased mitochondrial amounts of 3-MST and suggest that the H_2_S produced from this enzyme, in addition to the indisputable role of CBS, may contribute to the suppression of mitochondrial bioenergetics and the inhibition of cell proliferation in Down syndrome.

## Figures and Tables

**Table 1:** Description and origin of the human dermal fibroblasts (control cells: CC and Down syndrome cells: DSC) used in the present study.

Reference ID	Group ID	Origin	Description	Gender	Age at Sampling
DETROIT 551	CC	LGC STANDARDS	DIPLOID	FEMALE	FETUS
GM08447	CC	CORIELL INSTITUTE	DIPLOID	FEMALE	NEWBORN
CCD1064SK	CC	LGC STANDARDS	DIPLOID	MALE	NEWBORN
GM05756	CC	CORIELL INSTITUTE	DIPLOID	MALE	2 MONTHS
GM00041	CC	CORIELL INSTITUTE	DIPLOID	FEMALE	5 MONTHS
GM05659	CC	CORIELL INSTITUTE	DIPLOID	MALE	12 MONTHS
3-FCYPR10000286	CC	JéRôME LEJEUNE INSTITUTE	DIPLOID	MALE	5 YEARS
1-FCYPR10000368	CC	JéRôME LEJEUNE INSTITUTE	DIPLOID	FEMALE	12 YEARS
GM04616	DSC	CORIELL INSTITUTE	TRISOMY 21	FEMALE	NEWBORN
DETROIT 532	DSC	LGC STANDARDS	TRISOMY 21	MALE	2 MONTHS
GM02571	DSC	CORIELL INSTITUTE	TRISOMY 21	FEMALE	3 MONTHS
AG07096	DSC	CORIELL INSTITUTE	TRISOMY 21	MALE	5 MONTHS
AG05397	DSC	CORIELL INSTITUTE	TRISOMY 21	MALE	1 YEAR
DETROIT 539	DSC	LGC STANDARDS	TRISOMY 21	FEMALE	2 YEARS
3-FCYPR10000285	DSC	JéRôME LEJEUNE INSTITUTE	TRISOMY 21	MALE	5 YEARS
3-FCYPR10000369	DSC	JéRôME LEJEUNE INSTITUTE	TRISOMY 21	FEMALE	9 YEARS
